# A population‐adjusted indirect comparison of cardiovascular benefits of once‐weekly subcutaneous semaglutide and dulaglutide in the treatment of patients with type 2 diabetes, with or without established cardiovascular disease

**DOI:** 10.1002/edm2.259

**Published:** 2021-05-15

**Authors:** Lyndon Marc Evans, Linda Mellbin, Pierre Johansen, Jack Lawson, Abby Paine, Anna Sandberg

**Affiliations:** ^1^ Cardiff and Vale University Cardiff UK; ^2^ Department of Medicine Solna Karolinska Institutet Stockholm Sweden; ^3^ Novo Nordisk A/S Søborg Denmark; ^4^ Zedediah Consulting on behalf of DRG Abacus (part of Clarivate) Wokingham UK; ^5^ Present address: Novo Nordisk North West Europe Pharmaceuticals A/S Copenhagen Denmark; ^6^ Present address: Oncopeptides AB Luntmakargatan 46 Stockholm Sweden

**Keywords:** cardiovascular risks, GLP‐1 receptor agonist, type 2 diabetes

## Abstract

**Introduction:**

Cardiovascular (CV) effects of once‐weekly subcutaneous (s.c.) semaglutide 0.5 and 1 mg and dulaglutide 1.5 mg are reported in their respective placebo‐controlled cardiovascular outcome trials (CVOTs), SUSTAIN 6 and REWIND. There is no head‐to‐head CVOT comparing these treatments and heterogeneity between their CVOTs renders conventional indirect comparison inappropriate. Therefore, a matching‐adjusted indirect comparison (MAIC) was performed to compare the effects of s.c. semaglutide and dulaglutide on major adverse cardiovascular events (MACE) in patients with and without established cardiovascular disease (CVD).

**Methods:**

Individual patient data from SUSTAIN 6 were matched with aggregate data from REWIND, using a propensity score method to balance baseline effect‐modifying patient characteristics. Hazard ratios (HRs) for three‐point (3P) MACE (CV death, non‐fatal myocardial infarction, non‐fatal stroke), anchored via placebo, were then indirectly compared between balanced populations. Sensitivity analyses were performed to test the robustness of the main analysis.

**Results:**

After matching, included effect modifiers were balanced. In the main analysis, s.c. semaglutide was associated with a statistically significant 35% reduction in 3P MACE versus placebo (HR, 0.65 [95% confidence interval [CI]; 0.48, 0.87]) and nonsignificantly greater reduction (26%) versus dulaglutide (HR, 0.74 [95% CI; 0.54, 1.01]). Results were supported by all sensitivity analyses.

**Conclusions:**

This study demonstrated a statistically significant lower risk of 3P MACE for s.c. semaglutide versus placebo, in a population with lower prevalence of pre‐existing CVD than that in the pre‐specified primary analysis in SUSTAIN 6. Reduction in 3P MACE with s.c. semaglutide was greater than with dulaglutide, although not statistically significant.

## Graphical abstract

In the absence of head‐to‐head cardiovascular (CV) outcome trials comparing semaglutide and dulaglutide, the aim of this study was to compare the relative effect of these treatments on major adverse cardiovascular events (MACE) using a matching‐adjusted indirect comparison. Reduction in 3‐point MACE was greater with semaglutide versus dulaglutide, although not statistically significant in the population of patients with and without prior CV disease.

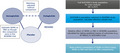

## 1 INTRODUCTION

Type 2 diabetes (T2D) is a chronic and progressive metabolic disorder associated with an elevated risk of microvascular and macrovascular complications, including cardiovascular disease (CVD), which can result in considerable morbidity and mortality.^1^, ^2^, ^3^, ^4^ Previous studies have shown that, while the effect of intensive blood glucose control decreases the risk of microvascular complications after a median of 5 years of follow‐up,^5^ its effect on macrovascular complications is only observed in the longer term for some cardiovascular (CV) outcomes.^6^ However, more recently, some glucose‐lowering medication classes have demonstrated significant CV benefit versus placebo in far shorter timeframes in their cardiovascular outcomes trials (CVOTs). These include glucagon‐like peptide receptor agonists (GLP‐1 RAs) and sodium‐glucose co‐transporter‐2 inhibitors (SGLT‐2is).^7^

For patients with T2D who have established CVD or indicators of high risk of CVD, GLP‐1 RAs and SGLT‐2is are recommended by the American Diabetes Association (ADA) and European Association for the Study of Diabetes (EASD), European Society of Cardiology and American College of Cardiology.^3^, ^8^, ^9^ However, based on findings of CVOTs, the ADA and EASD recommend GLP‐1 RAs as the preferred option when atherosclerotic CVD predominates and SGLT‐2is as the preferred option when heart failure (HF) or chronic kidney disease predominates.^8^ As well as differences between treatment classes, previous analyses suggest that CV benefit may vary within treatment class.^7^, ^10^ There are currently no head‐to‐head randomized controlled trials (RCTs) comparing CV benefit within treatment classes, and, in the absence of Food and Drug Administration (FDA) guidance on a standardized approach to the design of CVOTs, differences in study design between some CVOTs can make indirect comparison challenging. Robust, within‐class comparison could help to guide decisions on which product in a treatment class should be used to treat individual patients with T2D with CVD or CV risk factors.

Guidelines from the ADA and EASD specify that the GLP‐1 RA products used to treat patients with T2D and established CVD or at high risk of CVD should have proven CVD benefit, defined as having a label indication of reducing CVD events.^8^ In early 2020, two GLP‐1 RAs with once‐weekly dosing regimens, subcutaneous (s.c.) semaglutide and dulaglutide, were both approved by the FDA in this indication.^11^, ^12^ The CV effects of s.c. semaglutide were assessed in SUSTAIN 6, which demonstrated a statistically significant 26% reduction in the risk of three‐point (3P) major adverse cardiovascular events (MACE) (CV death, non‐fatal myocardial infarction [MI] or non‐fatal stroke) versus placebo in patients with T2D with established CVD and/or CV risk factors.^13^ The CV effects of dulaglutide were assessed in REWIND, which reported a statistically significant 12% reduction in the risk of 3P MACE with dulaglutide versus placebo with established CVD and/or CV risk factors.^14^ In the absence of head‐to‐head data comparing s.c. semaglutide with dulaglutide, an indirect comparison of these treatments based on their respective CVOTs could help to determine the most suitable GLP‐1 RA for patients with T2D at high CV risk.

Network meta‐analysis (NMA) is a well‐established method for conducting indirect treatment comparisons in the absence of head‐to‐head trials between treatments. Recently published NMAs have compared CVOTs to assess the effect of glucose‐lowering drugs on CV outcomes.^10^, ^15^, ^16^, ^17^ However, NMA adopts assumptions of homogeneity and similarity to provide unbiased estimates of treatment effects, and there must be no relevant heterogeneity between trials, which must have similar study designs, patient populations and outcome measures, and must be comparable on effect modifiers.^18^, ^19^ When significant heterogeneity exists between trials, NMA is rendered inappropriate. Alternative indirect comparative methods are available that seek to overcome heterogeneity, and the choice of an appropriate method will be determined by the type of evidence available, as described by Lingvay et al, 2020.^20^ Matching‐adjusted indirect comparison (MAIC) is an alternative method that can be used when individual patient data (IPD) are available for a treatment of interest and only published aggregate data (collated by treatment arm) are available for the comparator. MAIC addresses differences in patient populations using a propensity score‐based approach, which can provide a less biased estimate by weighting the IPD for an index treatment to match the aggregate baseline characteristics for a comparator.^21^, ^22^, ^23^

In an unpublished NMA feasibility analysis for comparison of GLP‐1 RAs, substantial heterogeneity was identified between CVOTs for s.c. semaglutide and dulaglutide in terms of patient baseline characteristics. Patients enrolled in SUSTAIN 6 were more likely to have experienced a prior CV event than those enrolled in REWIND, with approximately twice the proportion of patients experiencing prior ischaemic stroke (11.6% vs. 5.3%, respectively) and/or prior MI (32.5% vs. 16.2%, respectively). As such, an NMA was deemed unsuitable for comparing these CVOTs.

Therefore, with the availability of IPD from SUSTAIN 6 and aggregate data from REWIND, a MAIC was performed. The objective was to assess CV outcomes with s.c. semaglutide in the population with fewer prior CV events as assessed in REWIND and to indirectly compare the relative effects of s.c. semaglutide versus dulaglutide on rates of 3P MACE for patients with T2D with or without established CVD.

## 2 METHODS

### 2.1 Overview of the MAIC methodology

The MAIC approach was first published in 2010^23^ and has subsequently been described in detail in a number of publications, including guidance published in 2016 by the National Institute for Health and Care Excellence (NICE) Decision Support Unit (DSU) in their Technical Support Document (TSD) 18.^21^ The NICE guidance was accompanied by published code for use with the statistical package R,^24^ to enable MAIC to be carried out according to the recommendations set out in TSD 18 (Appendix D of the publication). Within the therapeutic area of diabetes, the MAIC approach has previously been used to compare the efficacy of two treatments within the same treatment class (dipeptidyl peptidase‐4 inhibitors) in a specific patient population.^25^ Further details of the MAIC methodology are provided in the Supporting Information.

### 2.2 Empirical approach and model specification

The methods used in the current study align with the NICE guidance.^21^ A systematic literature review (SLR) for interventions studied in CVOTs was conducted, alongside the unpublished NMA feasibility assessment, with a particular focus on GLP‐1 RA comparators. RCTs identified as relevant for the key treatments of interest were SUSTAIN 6,^13^ for which IPD were available, and REWIND^14^ with aggregate data. The 0.5 and 1 mg doses of s.c. semaglutide from the SUSTAIN 6 trial were pooled, as were the matching placebo arms, as the interest was in outcomes associated with s.c. semaglutide, not the specific doses. This increased the potential pool of patient data for s.c. semaglutide and was consistent with the results presented in the SUSTAIN 6 publication. These trials had a common comparator in placebo and thus an anchored MAIC could be conducted. PIONEER 6^26^ was also identified as a CVOT for which a different formulation of semaglutide (for once‐daily oral administration) was reported. Although not included in the main analysis, PIONEER 6 was included in a sensitivity analysis to compare the CV effects of the semaglutide molecule with dulaglutide.

Patient populations in SUSTAIN 6 and REWIND were similar in terms of age, gender and race (Table 1). However, patients in SUSTAIN 6 had a longer duration of diabetes than those in REWIND and a higher baseline HbA_1c_, with higher proportions of patients receiving insulin therapy. In addition, SUSTAIN 6 included higher proportions of patients with a history of CVD than REWIND, along with higher proportions of patients with some CV risk factors (estimated glomerular filtration rate [eGFR] <60 ml/min/1.73 m^2^ and albuminuria [urinary albumin‐to‐creatinine ratio (UACR) ≥3.39 mg/mmol]).

**TABLE 1:** Baseline characteristics of patients enrolled in SUSTAIN 6 and REWIND

	SUSTAIN 6		REWIND	
	Semaglutide 0.5 and 1 mg (*n* = 1648)	Placebo (*n* = 1649)	Dulaglutide 1.5 mg (*n* = 4949)	Placebo (*n* = 4952)
Age, years, mean (SD)	64.7 (7.2)	64.6 (7.5)	66.2 (6.5)	66.2 (6.5)
Gender, *n* (%)				
Female	635 (38.5)	660 (40.0)	2306 (46.6)	2283 (46.1)
Male	1013 (61.5)	989 (60.0)	2643 (53.4)	2669 (53.9)
Race, *n* (%)				
White	1384 (84.0)	1352 (82.0)	3754 (75.9)	3744 (75.6)
History of CVD, *n* (%)				
CVD	1262 (76.6)^a^	1271 (77.0)^a^	1560 (31.5)^b^	1554 (31.4)^b^
CV event	673 (40.8)^c^	694 (42.1)^c^	1028 (20.8)^d^	1007 (20.3)^d^
Previous HF	290 (17.6)	299 (18.1)	421 (8.5)	432 (8.7)
CV risk factors				
Current tobacco use, *n* (%)	204 (12.4)	202 (12.2)	694 (14.0)	713 (14.4)
Hypertension, *n* (%)	1543 (93.6)	1516 (91.9)	4605 (93.0)	4619 (93.3)
SBP, mmHg, mean (SD)	136.0 (17.47)	135.3 (16.82)	137.1 (16.6)	137.3 (17.0)
DBP, mmHg, mean (SD)	76.99 (10.00)	77.10 (10.04)	78.4 (9.8)	78.5 (9.9)
LDL cholesterol, mmol/L, mean (SD)	2.32 (0.95)	2.33 (0.99)	2.56 (0.98)	2.56 (0.98)
eGFR <60 ml/min/1.73 m^2^, *n* (%)	455 (27.6)	450 (27.3)	1081 (21.8)	1118 (22.6)
Albuminuria, *n* (%)	668 (41.3)	636 (39.2)	1707 (34.5)	1760 (35.5)
T2D				
Duration of diabetes, years, mean (SD); median (IQR)	14.2 (8.2); 13.2 (8.2–18.6)	13.6 (8.0); 12.6 (7.4–18.5)	10.5 (7.3); 9.5 (5.5–14.5)	10.6 (7.2); 9.5 (5.5–14.5)
HbA_1c_, %, mean (SD)	8.7 (1.5)	8.7 (1.5)	7.3 (1.1)	7.4 (1.1)
Change from baseline in HbA_1c_, %, mean	−1.25	−0.40	−0.46	−0.16
Glucose‐lowering drugs, *n* (%)				
Metformin	1211 (73.5)	1206 (73.1)	4022 (81.3)	4015 (81.1)
Sulphonylurea	698 (42.4)	712 (43.2)	2270 (45.9)	2282 (46.1)
Insulin	956 (58.0)	957 (58.0)	1189 (24.0)	1174 (23.7)
DPP‐4i	3 (0.1)	2 (0.1)	266 (5.4)	298 (6.0)
TZD	35 (2.1)	41 (2.5)	100 (2.0)	68 (1.4)
SGLT‐2i	1 (0.06)	4 (0.2)	2 (<0.1)	1 (<0.1)
CV medication, *n* (%)				
ACE inhibitor	829 (50.3)	813 (49.3)	2452 (49.5)	2463 (49.7)
ARB	548 (33.3)	563 (34.1)	1679 (33.9)	1693 (34.2)
β‐blocker	934 (56.7)	960 (58.2)	2237 (45.2)	2274 (45.9)
Calcium‐channel blocker	519 (31.5)	536 (32.5)	NR	NR
Other anti‐hypertensive	123 (7.5)	135 (8.2)	2767 (55.9)	2833 (57.2)
Statin	1199 (72.8)	1200 (72.8)	3279 (66.3)	3268 (66.0)
Fibrate	184 (11.2)	163 (9.9)	452 (9.1)	446 (9.0)
Platelet aggregation inhibitors	1200 (72.8)	1209 (73.3)	2662 (53.8)	2680 (54.1)

Abbreviations: ACE, angiotensin‐converting enzyme; ARB, angiotensin II receptor blocker; CV, cardiovascular; CVD, cardiovascular disease; DBP, diastolic blood pressure; DPP‐4i, dipeptidyl peptidase‐4 inhibitors; eGFR, estimated glomerular filtration rate; HbA_1c_, glycated haemoglobin; HF, heart failure; IQR, interquartile range; LDL, low‐density lipoprotein; MI, myocardial infarction; NR, not reported; SBP, systolic blood pressure; SD, standard deviation; SGLT‐2i, sodium‐glucose co‐transporter‐2 inhibitor; T2D, type 2 diabetes; TZD, thiazolidinedione.

^a^ Stroke, ischaemic heart disease (including myocardial infarction), peripheral arterial disease, ≥50% arterial stenosis in any artery, coronary revascularization (percutaneous coronary intervention or coronary artery bypass graft) or HF.

^b^ MI, ischaemic stroke, unstable angina with electrocardiogram changes, myocardial ischaemia on imaging or stress test, or coronary, carotid or peripheral revascularization.

^c^ MI or ischaemic, haemorrhagic or undetermined stroke.

^d^ MI or ischaemic stroke.

Effect‐modifying variables are not well established for CV outcomes in patients with T2D. Therefore, to enable matching in the analysis, potential effect modifiers were identified using input from clinical experts. For the purpose of this study, potential effect modifiers were identified as prior HF, prior MI, prior stroke or transient ischaemic attack (TIA), peripheral arterial disease (PAD), eGFR and albuminuria. Of these, prior MI, prior stroke or TIA and existing albuminuria were also described as ‘clinically relevant baseline characteristics for the primary endpoint’ in the REWIND trial publication.^14^ The six potential effect modifiers were considered to allow evaluation of the effect of GLP‐1 RAs across the spectrum of CV risk. In SUSTAIN 6 and REWIND, all variables were specified as dichotomous variables, that is, as a proportion of the patients in the trial with or without the condition at baseline. The exceptions were eGFR and UACR. In the primary REWIND publication, for eGFR, patients’ renal function was categorized as normal/mild (eGFR ≥60 ml/min/1.73 m^2^) and moderate/severe (eGFR <60 ml/min/1.73 m^2^) (dichotomous data), as well as mean and standard deviation (SD) measurements (continuous data) from the exploratory renal analysis publication.^27^ Similarly, for UACR, both the proportion of patients with albuminuria (dichotomous data) and the median and interquartile range (IQR) were available from the REWIND publications. Other risk factors, including diabetes duration, HbA_1c_ at baseline and smoking status, were not adjusted for in the model as these were considered to be prognostic and not effect‐modifying factors. In an anchored MAIC, provided prognostic factors are balanced between study arms, it is recommended not to adjust for them in the model since this can lead to loss of precision in the estimate of the relative treatment effect.^21^

**FIGURE 1:**
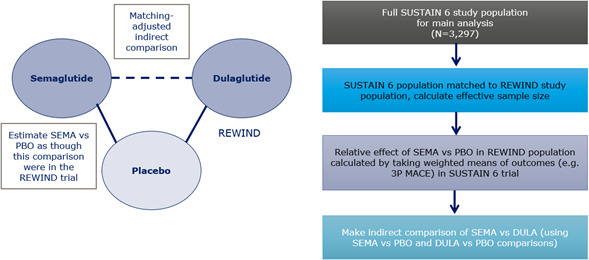
Summary of step‐by‐step MAIC process for the main analysis. Abbreviations: 3P MACE, 3‐point major adverse cardiovascular event; DULA, dulaglutide; PBO, placebo; SEMA, s.c. semaglutide

In the main analysis, summarized in Figure 1, IPD from SUSTAIN 6 were matched to the aggregate effect modifier baseline data from REWIND, based on matching all identified potential effect modifiers, with eGFR and albuminuria categorized as dichotomous variables (<60 vs. ≥60 ml/min/1.73 m^2^ and <3.39 vs. ≥3.39 mg/mmol, respectively). The matching for potential effect modifiers was achieved by running the relevant portion of the published NICE code in R (version 3.5.3). Thus, the s.c. semaglutide IPD were weighted to match the dulaglutide baseline characteristics using a form of propensity score model.^21^, ^22^ The weighting was calculated from the relevant baseline characteristic covariates only and was therefore independent of the outcome.

Details of the form of the propensity score modelling and weighting calculations can be found in the Supporting Information.

### 2.3 Outcomes of interest

Three‐point MACE was chosen as the primary outcome of interest as this was the primary endpoint in the identified trials. It was defined as first occurrence of death from CV causes (including undetermined death), non‐fatal MI or non‐fatal stroke. The published NICE code was modified to be suitable for use with time‐to‐event analyses such that, following matching, an adjusted hazard ratio (HR) for s.c. semaglutide versus placebo for 3P MACE was estimated in the target REWIND population. This was achieved by applying the calculated s.c. semaglutide patient weightings to the corresponding 3P MACE patient outcomes in a weighted Cox regression. The standard errors for the estimates were calculated using a robust sandwich estimator. The relative treatment effect of s.c. semaglutide and dulaglutide in the REWIND population could then be indirectly calculated using the HR for s.c. semaglutide versus placebo calculated in the first step, along with the HR reported in the REWIND publication for dulaglutide versus placebo.^14^

### 2.4 Sensitivity analyses

Three sensitivity analyses were performed to test the robustness of the results. The motivation for these was to explore the impact of the choice of effect modifiers and to examine the action of the semaglutide molecule, regardless of formulation.

For the first sensitivity analysis (sensitivity analysis 1), IPD from the SUSTAIN 6 and PIONEER 6 trials were used to calculate an adjusted HR value for semaglutide versus placebo in the population matched to the REWIND patient characteristics. Identical MAICs were used to match each semaglutide trial population separately to the REWIND data. The estimated HRs from both MAICs were then pooled using standard meta‐analysis techniques to estimate a single HR for the semaglutide molecule compared with placebo, matched to the REWIND trial. This was in accordance with the recommended approach in the NICE guidance, which proposes matching trials separately as a better approach than simply pooling the IPD and treating it as one large population in a single MAIC calculation.^21^ As in the main analysis, this pooled HR could then be used along with the HR from REWIND to indirectly compare the semaglutide molecule with dulaglutide.

Another sensitivity analysis (sensitivity analysis 2), which used IPD from SUSTAIN 6 only as in the main analysis, explored the choice of potential effect modifiers with re‐classification of eGFR and UACR data to balance the mean value and SD for eGFR as a continuous variable and the mean and SD of the log UACR. The log scale was chosen for UACR because only median and IQR were available from the published REWIND data, suggesting some skew in the data. Consequently, an estimation of mean and SD was first calculated on the natural scale from the median and IQR using the methods proposed by Wan et al, 2014^28^ and then the log values calculated for matching.

The final sensitivity analysis (sensitivity analysis 3) also used IPD from SUSTAIN 6 only and considered exclusion of baseline kidney function factors (eGFR and albuminuria) entirely.

## 3 RESULTS

### 3.1 Baseline characteristic matching

For the main analysis, all six identified potential effect modifiers were included, with both eGFR and albuminuria measured as dichotomous variables (<60 vs. ≥60 ml/min/1.73 m^2^ and UACR <3.39 vs. ≥3.39 mg/mmol, respectively). A histogram showing the distribution of the re‐scaled weightings for each individual patient in the SUSTAIN 6 trial is provided in the Supporting Information.

A comparison of the adjusted potential effect‐modifying baseline patient characteristics in the SUSTAIN 6 and REWIND trials before and after matching is presented in Table 2. After matching, the characteristics were exactly balanced between the trials, with the effective sample sizes of the population in SUSTAIN 6, a measure of the patient overlap between trials, being approximately 20% smaller than the original trial data (Table 2). Results of the matching of PIONEER 6 baseline data for the sensitivity analysis are presented in the Supporting Information.

**TABLE 2:** Comparison of effect‐modifying baseline patient characteristics from SUSTAIN 6 and REWIND trials before and after matching

Baseline characteristics	SUSTAIN 6		REWIND
	s.c. SEMA vs. PBO		DULA vs. PBO
	Before matching (*N* = 3297)	After matching (ESS = 2633)	As reported (*N* = 9901)
Prior HF (NYHA II–III)	17.9% (*n* = 589)	8.6% (*n* = 226)	8.6%^a^ (*n* = 853)
Prior stroke or TIA	12.1% (*n* = 400)	9.1% (*n* = 240)	9.1% (*n* = 899)
Prior MI	32.5% (*n* = 1072)	16.2% (*n* = 427)	16.2% (*n* = 1602)
Prior PAD	14.0% (*n* = 460^b^)	8.7% (*n* = 229)	8.7% (*n* = 856)
eGFR <60 ml/min/1.73 m^2^	27.4% (*n* = 905^c^)	22.2% (*n* = 585)	22.2% (*n* = 2199)
Albuminuria, UACR ≥3.39 mg/mmol	40.3% (*n* = 1329)	35.0% (*n* = 922)	35.0% (*n* = 3467)

Note

*N* = total number of randomized patients; *n* = number of patients with prior event across all treatment arms.

Abbreviations: DULA, dulaglutide; eGFR, estimated glomerular filtration rate; ESS, effective sample size; HF, heart failure; MI, myocardial infarction; NYHA, New York Heart Association; PAD, peripheral arterial disease; PBO, placebo; s.c., subcutaneous; SEMA, semaglutide; TIA, transient ischaemic attack; UACR, urinary albumin‐to‐creatinine ratio.

^a^ NYHA stage unclear at time the analysis was conducted; stage II–III was chosen for matching from SUSTAIN 6 as this was considered a more conservative approach, that is the difference in proportions of patients with prior HF between trials with I‐III would have been even wider than the 17.9% versus 8.6%.

^b^ Includes 7 patients with >50% stenosis in peripheral arteries on angiography or imaging, but no prior diagnosis of PAD.

^c^ At baseline, compared with values at screening reported in (Marso et al.^13^), *n* = 939.

### 3.2 Estimated relative treatment effect on 3P MACE—results of the main analysis

In the main analysis, following the re‐weighting of the observed 3P MACE patient outcomes in SUSTAIN 6, s.c. semaglutide was associated with a statistically significant 35% reduction in 3P MACE compared with placebo (HR, 0.65 [95% CI; 0.48, 0.87]) (Table 3). The relative treatment effect of dulaglutide versus placebo was taken from the REWIND trial (HR, 0.88 [95% CI; 0.79, 0.99], *p *= .026), and this allowed the indirect comparison between s.c. semaglutide and dulaglutide to be calculated, resulting in a nonsignificantly greater reduction (26%) with s.c. semaglutide compared with dulaglutide (HR, 0.74 [95% CI; 0.54, 1.01]).

**TABLE 3:** MAIC results of main analysis. Comparison of relative treatment effect on 3P MACE

Comparison	Before matching, as published, HR (95% CI)	After matching	
		HR (95% CI)	*p*‐value
s.c. SEMA vs. PBO	0.74 (0.58, 0.95)	0.65 (0.48, 0.87)	.004
s.c. SEMA vs. DULA	N/A	0.74 (0.54, 1.01)	.06

Abbreviations: 3P MACE, 3‐point major adverse cardiovascular event; CI, confidence interval; DULA, dulaglutide; HR, hazard ratio; MAIC, matching‐adjusted indirect comparison; N/A, not applicable; PBO, placebo; s.c., subcutaneous; SEMA, semaglutide.

### 3.3 Sensitivity analyses results

In sensitivity analysis 1, when IPD from the SUSTAIN 6 and PIONEER 6 trials were matched separately to the REWIND data and outcomes pooled using meta‐analysis to estimate a single HR value for the semaglutide molecule (subcutaneous and oral formulations) versus dulaglutide, semaglutide demonstrated a statistically significant reduction in 3P MACE compared with dulaglutide (HR, 0.76 [95% CI; 0.58, 0.99]; *p *= .04) (Table 4).

**TABLE 4:** MAIC sensitivity analyses. Comparison of relative effect on 3P MACE

Comparison	Before matching, as published, HR (95% CI)	After matching with REWIND trial	
		HR (95% CI)	*p*‐value
Sensitivity analysis 1 (S6 + P6, all EMs, eGFR and UACR dichotomous^a^)			
SEMA vs. PBO	0.76 (0.62, 0.92)	0.67 (0.53, 0.85)	*p *< .001
SEMA vs. DULA	N/A	0.76 (0.58, 0.99)	.04
Sensitivity analysis 2 (S6, all EMs, eGFR and UACR continuous)			
s.c. SEMA vs. PBO	0.74 (0.58, 0.95)	0.68 (0.50, 0.93)	.01
s.c. SEMA vs. DULA	N/A	0.78 (0.56, 1.07)	.11
Sensitivity analysis 3 (S6, CVD EMs only)			
s.c. SEMA vs. PBO	0.74 (0.58, 0.95)	0.67 (0.50, 0.90)	.007
s.c. SEMA vs. DULA	N/A	0.76 (0.56, 1.04)	.09

Abbreviations: 3P MACE, 3‐point major adverse cardiovascular event; CI, confidence interval; CVD, cardiovascular disease; DULA, dulaglutide; eGFR, estimated glomerular filtration rate; EM, effect modifier; HR, hazard ratio; MAIC, matching‐adjusted indirect comparison; N/A, not applicable; P6, PIONEER 6; PBO, placebo; s.c., subcutaneous; S6, SUSTAIN 6; SEMA, semaglutide; UACR, urinary albumin‐to‐creatinine ratio.

^a^ As eGFR <60/eGFR ≥60 ml/min/1.73 m^2^ and UACR <3.39/UACR ≥3.39 mg/mmol.

**FIGURE 2:**
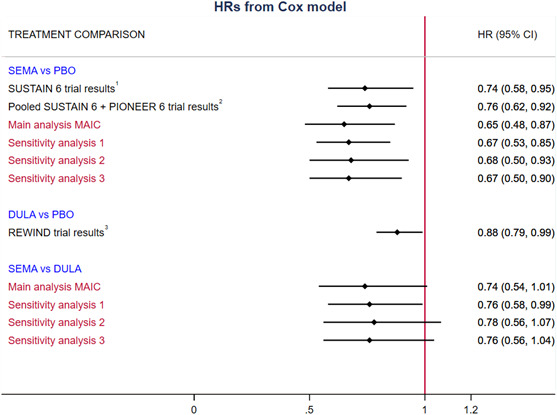
Forest plot for matching IPD from semaglutide trials with REWIND aggregate data—3P MACE^†^ Abbreviations: 3P MACE, 3‐point major adverse cardiovascular event; CI, confidence interval; CVD, cardiovascular disease; DULA, dulaglutide; eGFR, estimated glomerular filtration rate; HF, heart failure HR, hazard ratio; IPD, individual patient data; MAIC, matching‐adjusted indirect comparison; MI, myocardial infarction; PAD, peripheral arterial disease; PBO, placebo; SEMA, semaglutide; UACR, urinary albumin‐to‐creatinine ratio. ^†^Sensitivity analysis 1: SUSTAIN 6 + PIONEER 6, CVD effect modifiers (MI, stroke, HF, PAD), eGFR and UACR dichotomous (eGFR <60/eGFR ≥60 ml/min/1.73 m^2^; UACR <3.39 vs. ≥3.39 mg/mmol); Sensitivity analysis 2: SUSTAIN 6, CVD effect modifiers (MI, stroke, HF, PAD), eGFR and UACR continuous; Sensitivity analysis 3: SUSTAIN 6, CVD effect modifiers (MI, stroke, HF, PAD). References: 1. (Marso et al.^13^); 2. (Husain et al.^13^); 3. (Gerstein et al.^13^)

In sensitivity analysis 2, which explored the impact of reclassifying eGFR and UACR data as continuous rather than dichotomous data, and sensitivity analysis 3, which excluded eGFR and albuminuria data entirely, the mean HR values for s.c. semaglutide versus placebo and versus dulaglutide were comparable with those estimated in the main analysis (Table 4). A forest plot showing all the relative treatment effects after matching IPD from semaglutide trials with REWIND aggregate data is presented in Figure 2.

## 4 DISCUSSION

This study compared the relative effect of two GLP‐1 RAs (s.c. semaglutide vs. dulaglutide) on rates of 3P MACE (CV death, non‐fatal MI, or non‐fatal stroke) in patients with T2D with or without established CVD, using a MAIC approach.

The main analysis showed that, compared with placebo, s.c. semaglutide was associated with a statistically significant 35% reduction in 3P MACE in a population with a lower prevalence of pre‐existing CVD than that enrolled in the SUSTAIN 6 trial. This adjusted reduction was greater than that demonstrated in the pre‐specified primary analysis from SUSTAIN 6 in which s.c. semaglutide was associated with a 26% reduction in 3P MACE compared with placebo. The main analysis also indicated that s.c. semaglutide resulted in at least as great a reduction in the risk of 3P MACE as dulaglutide, with a nonsignificantly lower risk of events as shown by the point estimate for the relative treatment effect, which was <1.0 (HR 0.74, [95% CI; 0.54, 1.01]).

Results of the main analysis were supported by all sensitivity analyses conducted. In one of these sensitivity analyses, SUSTAIN 6 adjusted 3P MACE results for s.c. semaglutide were pooled with the corresponding results for PIONEER 6 for oral semaglutide to provide an estimate of treatment effect for the semaglutide molecule. In this analysis, statistically significant 33% and 24% reductions in 3P MACE were demonstrated for the semaglutide molecule compared with placebo and dulaglutide, respectively. Results from this MAIC are in line with those reported in a published pooled post hoc analysis of SUSTAIN 6 and PIONEER 6 IPD, which showed a statistically significant 24% reduction in 3P MACE for semaglutide versus placebo in patients with established CVD and/or CV risk factors.^29^

Although NMAs have previously been used to compare CVOTs to assess the effect of glucose‐lowering drugs on CV outcomes,^10^, ^15^, ^16^, ^17^ none of the published NMAs have incorporated measures to adjust for any differences between trials in their model, which may result in bias. Furthermore, with a sparse network of treatments consisting of just one or two trials per treatment, indirect comparisons are vulnerable to systematic bias resulting from imbalances in effect modifier distributions.^21^

While some previous studies have compared CV effects between treatment classes, guidelines make recommendations regarding the treatment class to be used for particular patient groups.^8^ Therefore, the current study focuses on the comparison between two treatments of the same class, s.c. semaglutide and dulaglutide, two GLP‐1 RAs with once‐weekly subcutaneous administration and with label indications of reducing CV events in patients with T2D^11^, ^12^. This may help to improve understanding of the clinical differences between products within the GLP‐1 RA treatment class for patients with T2D at high CV risk. With availability of IPD from SUSTAIN 6 and published aggregate data from REWIND, the current study used a MAIC approach to overcome the heterogeneity between these trials that renders conventional indirect comparison methods unsuitable. This allowed comparison of 3P MACE for s.c. semaglutide and dulaglutide in a broad population of patients with or without established CVD. While the eligibility criteria employed in RCTs may limit generalizability to the population with T2D, this broad population may be considered more generalizable to patients with T2D in clinical practice than the SUSTAIN 6 population.^14^, ^30^

There are several strengths of the current study compared with analyses that have used a conventional NMA approach to assess the effect of anti‐diabetic drugs on CV outcomes. Findings from this study are robust because the MAIC approach implemented followed the NICE guidance methodology on population‐adjusted indirect comparisons.^21^ This MAIC has shown that incorporating IPD from trials of one treatment into indirect comparisons can help to address several limitations that apply to analyses based only on published aggregate data. By balancing key effect‐modifying baseline characteristics across the SUSTAIN 6, PIONEER 6 (sensitivity analysis), and REWIND trials, this MAIC reduced the differences in potential effect‐modifying factors, providing less biased estimates of relative treatment effects compared with conventional indirect comparisons in this indication, which are limited by cross‐trial differences.^10^, ^15^, ^16^, ^17^ The strength of the anchored MAIC is that it uses a common comparator to balance prognostic variables within studies.

The main limitation of the current study is likely to be the selection of potential effect modifiers, which are not well established for CV outcomes in T2D. To overcome this, we selected potential effect modifiers based on clinical expertise and aimed to select those that would provide estimates of the effects of GLP‐1 RAs across the spectrum of CV risk. However, the choice of potential effect modifiers is, to some extent, subjective and it is anticipated that choosing different variables to match may alter the findings of the analyses. Furthermore, results could only be adjusted for reported aggregate data and corresponding data available from IPD records. Therefore, bias associated with differences in baseline characteristics between trials may remain. An additional limitation is the difference in follow‐up periods between the studies, with a median follow‐up time of 2.1 years in SUSTAIN 6, compared with 5.4 years in REWIND. It would be desirable to have an extended follow‐up period for SUSTAIN 6 to determine whether the treatment effect varies over time. Ideally, the findings of this study would be validated in a head‐to‐head RCT.

Despite these limitations, compared with conventional indirect comparison approaches, which would have been confounded by differences in baseline characteristics between the trials, this study allows for a more robust and objective comparison of the relative CV risk reduction with s.c. semaglutide versus dulaglutide in patients with or without established CVD (matched for prior HF, MI, stroke, PAD, eGFR and albuminuria).

This MAIC study demonstrated a statistically significant lower risk of 3P MACE for s.c. semaglutide versus placebo (population‐adjusted HR: 0.65) in a population with lower prevalence of pre‐existing CV disease (REWIND trial population) than that enrolled in the SUSTAIN 6 trial (pre‐specified primary analysis HR: 0.74). The analysis also indicated that s.c. semaglutide resulted in a nonsignificantly greater reduction versus dulaglutide in the risk of 3P MACE (indirect comparison estimate HR 0.74, *p *= .06) in this population. The findings of this study may help to improve understanding of the clinical differences between products within the GLP‐1 RA treatment class for patients with T2D at high CV risk.

## CONFLICTS OF INTEREST

J.L. is an employee of Novo Nordisk A/S. P.J. and A.S. were employees of Novo Nordisk A/S during development of the manuscript. A.P. received consultancy fees for the statistical analyses presented in this manuscript; she has also received personal fees from Novo Nordisk, Sanofi and Ultragenyx. L.M.E. has received lecture fees from AstraZeneca, Boehringer Ingelheim, Eli Lilly, and Novo Nordisk and research support from AstraZeneca and Novo Nordisk. Linda Mellbin has received lecture fees from Amgen, AstraZeneca, Bayer AB, Boehringer Ingelheim, Merck Sharp & Dohme, Novo Nordisk and Sanofi Aventis. She has also received consulting fees from or been involved in clinical trials funded by AstraZeneca, Bayer AG, Boehringer Ingelheim, Novo Nordisk and Sanofi Aventis.

## AUTHOR CONTRIBUTIONS

All authors contributed to the conception, drafting and critical editing of the manuscript. A.P. conducted the statistical analyses.

## Supporting information

Supplementary Material

## Data Availability

Research data not shared.
