# Supplementary material for: A population‐adjusted indirect comparison of cardiovascular benefits of once‐weekly subcutaneous semaglutide and dulaglutide in the treatment of patients with type 2 diabetes, with or without established cardiovascular disease
Source: Endocrinol Diabetes Metab. 2021 May 15;4(3):e00259. doi: 10.1002/edm2.259 (PMC8279621; doi:10.1002/edm2.259)
Supplement: Supplementary file 1 — Supplementary Material [file EDM2-4-e00259-s001.docx]

## Supporting information

**Overview of MAIC methodology**

In the application of MAIC, IPD available from trials of one treatment are matched to baseline summary statistics reported from trials of another treatment. A number of steps are necessary in this analysis. Firstly, as with any meta-analysis or other form of data synthesis, an SLR should identify all possible clinical trials for the treatments of interest (Signorovitch et al., 2012). Variables for adjustment can then be identified, either from clinical expertise or prior empirical evidence (Phillippo et al., 2016). The NICE guidance recommends that an anchored indirect comparison should always be preferred to unanchored comparisons where there is a common comparator, as it respects the randomisation within studies and fewer assumptions are required. The guidance also recommends that, for an anchored comparison, only effect modifiers should be adjusted for. These are covariates that alter the effect of treatment on outcomes, so that the treatment is more or less effective in different subgroups formed by different levels of the effect modifier. Purely prognostic factors (covariates that affect outcomes) should not be adjusted for, to avoid loss of precision due to over-matching (Phillippo et al., 2016).

Following effect modifier selection, a logistic propensity score model is used to calculate a weighting for each patient in the trial with the available IPD. This weighting reflects adjustment of the distribution of baseline characteristics to match those in the trial for which only published aggregate data are available. The propensity scores are estimated using a method of moments to match average effect-modifying baseline characteristics across the trials being compared (Phillippo et al., 2016). A measure of the amount of overlap between the study populations is the effective sample size (ESS) (Signorovitch et al., 2010). ESS is an adjustment of the IPD trial sample size that accounts for the weighting of the observations, and the resulting correlations between estimated responses. As with usual sample sizes, a larger ESS is preferable to a small ESS, as the larger sample has greater power to detect any potential differences between treatments.

Finally, these estimated weights are used to predict outcomes for each patient for which IPD are available, as though they had been enrolled in the aggregate data trial. Anchored indirect comparison estimates can then be created to compare the two treatments of interest.

**Equations used to implement the MAIC approach (Phillippo et al., 2016; Signorovitch et al., 2010)**

As with likelihood reweighting (from which MAIC is derived) the individual patient weights, $\omega_{i}$, are estimated using a logistic propensity score model, as follows:

$\log\left( \omega_{i} \right)= \alpha_{0}+ {\boldsymbol{\alpha}_{1}^{T}\boldsymbol{X}}_{it}$ (1)

for the *ith* patient, receiving treatment *t*, with covariates ***X****_it_* where *α* is a constant.

Due to the lack of IPD available from the REWIND trial, the regression parameters are not estimable using standard methods. Signorovitch (Signorovitch et al., 2010) proposed using a method of moments to exactly balance the covariate values and their variance, thus the weights are obtained by minimising

$\sum_{i,t} exp(\boldsymbol{\alpha}_{1}^{T}\boldsymbol{X}_{it}^{EM})$ (2)

where EM refers to the respective effect modifier.

The weights are rescaled relative to the unit weights of the original dataset based on sample size (N), which facilitates the interpretation of the distribution of weights:

$\tilde{\omega_{i}}= \frac{\omega_{i}N}{\sum_{i} \omega_{i}}$ (3)

as shown graphically in Figure 3 for the main analysis.

An estimate of the effective sample size (ESS) in the adjusted SUSTAIN 6 data can be obtained using the equation

$ESS= \frac{{(\sum_{t=1}^{T} \sum_{i=1}^{N} \omega_{it})}^{2}}{\sum_{t=1}^{T} \sum_{i=1}^{N} \omega_{it}^{2}}$ (4)

where $\omega_{it}$ is the weight for the *ith* patient receiving treatment *t*.

A simple weighted average outcome is defined as:

$\hat{Y_{(T)}}= \frac{\sum_{i=1}^{N} Y_{i(I)}\omega_{i}}{\sum_{i=1}^{N} \omega_{i}}$ (5)

where $\hat{Y_{(T)}}$ is the estimated mean outcome in the target population, $Y_{i(I)}$ is the observed outcome for individual *i* in the SUSTAIN 6 population.

For the current analysis, a weighted Cox model was implemented to account for the characteristic balancing. The standard form of the Cox regression model is given by the equation:

h(t) = h_0_(t) x exp(β_1_.x_i1_ + β _2_.x_i2_ + ... + β _k_.x_ik_)

where *h_0_(t)* is the baseline or underlying hazard function and corresponds to the instantaneous risk of having an event when all the explanatory variables are zero, *x_ik_* is the covariate vector and β*_k_* are the regression coefficients.

**Additional details of baseline characteristic matching**

The distribution of the re-scaled weightings for each individual patient in the SUSTAIN 6 trial is shown in Figure S1. A re-scaled weighting greater than 1.0 indicates that a patient is given more weight in the matched population than the original SUSTAIN 6 trial population. Conversely, a re-scaled weighting smaller than 1.0 indicates that a patient has been assigned a lower weighting than in the original trial. Individual patient weightings ranged between 0.08 and 1.77, with a median weight of 0.96.

Figure S1: Re-scaled weightings for SUSTAIN 6 patients matched with REWIND aggregate data – base case analysis


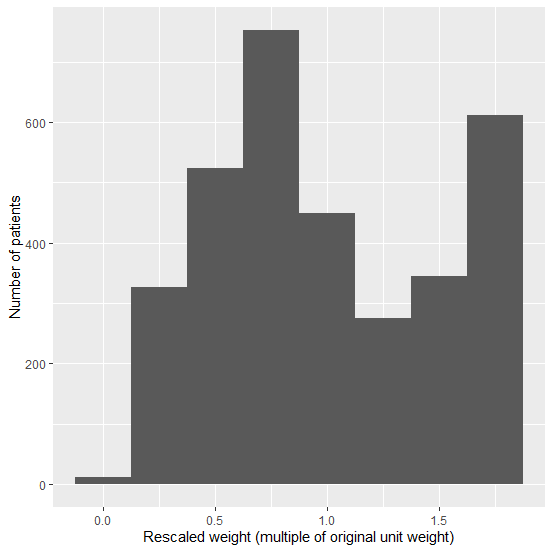


Figure legend: Each bar in the histogram represents a range of 0.25 in weighting; for example, the bar at 1.5 represents patients with a weighting between 1.375 and 1.625.

A comparison of the adjusted effect modifying baseline patient characteristics in the PIONEER 6 and REWIND trials before and after matching is presented in Table S1.

Table S1: Comparison of effect modifying baseline patient characteristics from PIONEER 6 and REWIND trials before and after matching

| **Baseline characteristics** | **PIONEER 6** | | **REWIND** |
| --- | --- | --- | --- |
|  | **Oral SEMA vs PBO** | | **DULA vs PBO** |
|  | **Before matching (N=3,183)** | **After matching (ESS=2,491)** | **As reported (N=9,901)** |
| **Prior HF (NYHA II-III)** | 12.2%  (n=388) | 8.6% (n=214) | 8.6%^†^ (n=853) |
| **Prior stroke or TIA** | 11.7% (n=374) | 9.1% (n=227) | 9.1% (n=899) |
| **Prior MI** | 36.1% (n=1,150) | 16.2% (n=404) | 16.2% (n=1,602) |
| **Prior PAD** | 8.2% (n=261) | 8.7% (n=217) | 8.7% (n=856) |
| **eGFR <60 mL/ min/1.73 m^2^** | 26.9% (n=856) | 22.2% (n=553) | 22.2% (n=2,199) |

Abbreviations: DULA, dulaglutide; eGFR, estimated glomerular filtration rate; ESS, effective sample size; HF, heart failure; MI, myocardial infarction; NYHA, New York Heart Association; PAD, peripheral arterial disease; PBO, placebo; SEMA, semaglutide; TIA, transient ischaemic attack.
N=total number of randomised patients; n=number of patients with prior event across all treatment arms
†NYHA stage unclear.

## References

Phillippo, D., Ades AE, Dias S, Palmer S, Abrams KR & Welton NJ (2016). *NICE DSU Technical support document 18: Methods for population-adjusted indirect comparisons in submissions to NICE.* [Online]. Available: <http://nicedsu.org.uk/wp-content/uploads/2017/05/Population-adjustment-TSD-FINAL.pdf> [Accessed].

Signorovitch, J. E., Sikirica, V., Erder, M. H., Xie, J., Lu, M., Hodgkins, P. S., Betts, K. A. & Wu, E. Q. (2012). Matching-adjusted indirect comparisons: a new tool for timely comparative effectiveness research. *Value Health,* **15**(6)**,** 940-7.

Signorovitch, J. E., Wu, E. Q., Yu, A. P., Gerrits, C. M., Kantor, E., Bao, Y., Gupta, S. R. & Mulani, P. M. (2010). Comparative effectiveness without head-to-head trials: a method for matching-adjusted indirect comparisons applied to psoriasis treatment with adalimumab or etanercept. *Pharmacoeconomics,* **28**(10)**,** 935-45.
